# Seroprevalence of brucellosis, Q fever and Rift Valley fever in domestic ruminants in Guinea in 2017–2019

**DOI:** 10.1186/s12917-022-03159-x

**Published:** 2022-02-04

**Authors:** Cécile Troupin, Isabelle Ellis, Bakary Doukouré, Alimou Camara, Moustapha Keita, Moise Kagbadouno, Jean-Mathieu Bart, Ramadan Diallo, Sandra Lacôte, Philippe Marianneau, Martin H Groschup, Noël Tordo

**Affiliations:** 1 Institut Pasteur de Guinée, BP 4416, Conakry, Guinea; 2 Present address: Institut Pasteur du Cambodge, Virology Unit, Phnom Penh, Cambodia; 3 Institut National de Santé Publique, Conakry, Guinea; 4 Institut Supérieur Des Sciences Et de Médecine Vétérinaire, Dalaba, Guinea; 5 Programme National de Lutte Contre La Trypanosomiase Humaine Africaine, Conakry, Guinea; 6 Umr Intertryp Ird-Cirad, Université de Montpellier, Montpellier, France; 7 Laboratoire Central de Diagnostic Vétérinaire, Conakry, Guinea; 8 ANSES, Lyon Laboratory, Virology Unit, Lyon, France; 9 Friedrich‐Loeffler‐Institut, Institute of Novel and Emerging Infectious Diseases, Greifswald Insel Riems, Greifswald, Germany

**Keywords:** Brucellosis, Q fever, Rift Valley fever, Serology, Domestic ruminants, Guinea

## Abstract

**Background:**

Brucellosis, Q fever and Rift Valley fever are considered as Neglected Zoonotic Diseases (NZDs) leading to socioeconomic losses in livestock globally, and particularly in developing countries of Africa where they are under-reported. In this study, we evaluated the seroprevalence of these 3 zoonotic diseases in domestic ruminants in Guinea from 2017 to 2019. A total of 1357 sera, sampled from 463 cattle, 408 goats and 486 sheep, were collected in 17 Guinean prefectures and analyzed by enzyme-linked immunosorbent assay (ELISA).

**Results:**

Cattle was the species with highest seroprevalence (5 to 20-fold higher than in small ruminants) for the three diseases. The seroprevalence of brucellosis, mostly focused in Western Guinea, was 11.0% (51 of 463) in cattle, 0.4% (2 in 486) in sheep while no specific antibodies were found in goats. Q fever, widespread across the country, was the most frequently detected zoonosis with a mean seroprevalence of 20.5% (95 in 463), 4.4% (18 in 408) and 2.3% (11 in 486) in cattle, goats and sheep, respectively. The mean seroprevalence of RVF was 16.4% (76 in 463) in cattle, 1.0% (4 in 408) in goats and 1.0% (5 in 486) in sheep. Among the samples 19.3% were seropositive for at least one of the three NZDs, 2.5% showed specific antibodies against at least two pathogens and 4 cattle (0.8%) were seropositive for all three pathogens. In cattle, adults over 3-years old and females presented a higher antibody seroprevalence for the three diseases, in congruence with putative exposure risk.

**Conclusions:**

This study confirms the circulation of these three zoonotic pathogens in Guinea and highlights the need for implementing a syndromic surveillance of ruminant abortions by the Guinean veterinary authorities as well as for the screening of the human population at risk (veterinarians, breeders, slaughterers) in a One Health perspective.

**Supplementary Information:**

The online version contains supplementary material available at 10.1186/s12917-022-03159-x.

## Introduction

More than two-third of the newly emerging infectious agents affecting humans are zoonotic in origin [1]. Neglected Zoonotic Diseases (NZDs) such as brucellosis, Q fever and Rift Valley fever (RVF) are under-diagnosed and under-reported in developing countries [2]. These three zoonoses cause abortions in cattle and small ruminants and can lead to important economic losses for the livestock husbandry worldwide and particularly in Africa [3].

Brucellosis is caused by gram-negative bacteria from the genus *Brucella*. *Brucella abortus* affects cattle, *Brucella melitensis* is found in small ruminants and *Brucella suis* in pigs. Transmission between animals mainly occurs by ingestion of infected birth fluids but also through cuts in the skin, or through mucous membranes. *Brucella* can cause fever, arthritis and neurological symptoms in humans [4, 5]. Brucellosis is endemic almost worldwide but infections in humans became rare after eradication programs with selective slaughtering of infected ruminants and by the introduction of food hygiene measures in Western and Northern Europe, Canada, United States, Japan, Australia and New Zealand [4]. In Africa, and particularly in the Sub-Saharan region, brucellosis is still endemic [6, 7] with a seroprevalence varying from 0 to 40% in human and livestock depending on the geographical location [8].

Q Fever is caused by the intracellular gram-negative bacterium *Coxiella burnetii* [9]. A variety of wild and domestic host species have been identified but domestic ruminants such as cattle, goats and sheep are the major source of human infections [10]. Q Fever is present worldwide except in New Zealand [11]. In Western and Central Africa, a high seroprevalence is observed in animals, 18–55% in cattle and 11–33% in small ruminants with significant production losses [12]. *C. burnetii* is shed in milk, urine and feces but most importantly, during parturition in amniotic fluids and placenta. Transmission can also occur by spore inhalation or by tick vectors between animals. In humans Q fever is mainly asymptomatic or provokes self-limited illness with flu-like symptoms and transient hepatitis and pneumonia. Rare cases progress into chronic infection with endocarditis or vascular infection which can be lethal [13]. In Africa, it accounts for 2–9% of febrile illness hospitalizations and 1–3% of endocarditis [12]. It can be misdiagnosed as malaria [14, 15].

Rift Valley fever (RVF) is a viral infection caused by a negative strand RNA virus belonging to the order *Bunyavirale*s, family *Phenuiviridae*, genus *Phlebovirus* [16]. Rift Valley fever virus (RVFV) infects wildlife, livestock and humans. In humans RVFV causes mostly mild disease, weakness, back pain and dizziness, although severe symptoms such as hemorrhages, meningoencephalitis can occur infrequently as well. Typical ocular sequelae (retinitis) with macular lesions are also seen more often [17, 18]. RVFV is an arbovirus transmitted by mosquitoes of several genera, including *Aedes spp*. and *Culex spp*. [19, 20]. Since their first description in 1931 in Kenya RVF cases have been reported only in Africa and the Arabic Peninsula [21, 22]. A systematic review performed on 126 articles over the last 5 decades reports that in Africa the seroprevalence of RVF varied geographically and temporally in livestock, wildlife and humans, ranging from 0 to 100% in cattle and sheep, from 0 to 69.6% in goats, from 0 to 57.1% in camels, from 0 to 87.5% in wildlife, and from 0 to 81.0% in humans [23]. The highest RVF seroprevalence in livestock was found during the 1997 epizootic episode in Egypt where 100% of the sheep and cattle samples were seropositive [24].

Guinea is situated in an intermediate position in Africa, connecting different ecosystems: a rather dry Sahelian area in the North in contact with Senegal and Mali, a wet forest area in the South in contact with Ivory Coast, Liberia and Sierra Leone, and the Atlantic littoral in the West. Only limited studies have been performed to evaluate the prevalence of brucellosis, Q fever and RVF in the country. The first recorded data of bovine brucellosis showed an overall seroprevalence of 6.9% (range 0–27%) depending on the geographical location [25]. Since then, several studies confirmed the influence of the geographical location with overall bovine seroprevalence ranging from 8.7% (range 5.3–12%) [26] to 11.8% [27]. For Q fever, the only published study reported *C. burnetii* circulation with a mean seroprevalence of 2.4% (range 0.8–10.5%) in humans and of 8.0% (range 3.2–18.7%) in livestock [28]. RVFV circulation was first reported in 1987 with the isolation of RVFV from 2 bats species and the detection of RVFV specific antibodies in 3.3% of human and 6.8% of domestic animal sera [29]. Another study performed in six Western African countries reported 5% of RVF seroprevalence in 40 bovines from Guinea [30]. More recent studies reported 0 to 7% seroprevalence of RVF in cattle depending on the geographical location in Guinea [27, 31].

Up to now, all seroprevalence studies in Guinea focused only on cattle and the circulation of all three pathogens was never investigated simultaneously on the same animals during the same period. The purpose of the present study was to generate updated epidemiological data in the three diseases on domestic ruminants (cattle, goats and sheep) in Guinea. Information on seroprevalence and risk factors associated with these three zoonoses will be useful to evaluate the associated risks on human health and animal production and to define suitable prevention and control programs in Guinea.

## Results

**Fig. 1:**
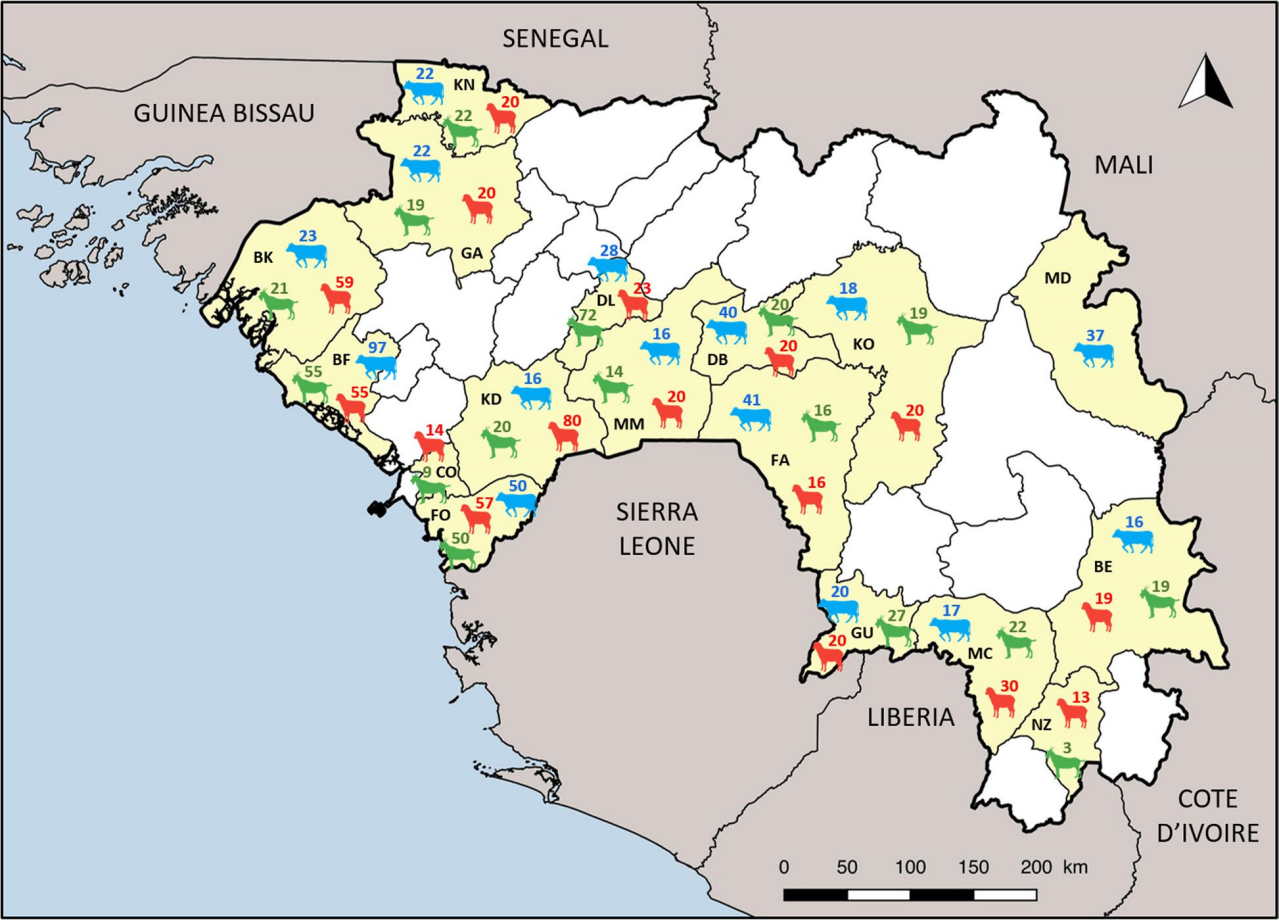
Map of blood samples collected in this study. The 17 prefectures where samples have been collected are indicated in yellow and their names are represented by a two-letters codes in black (Beyla (BE); Boffa (BF); Boke (BK); Coyah (CO); Dabola (DB); Dalaba (DL); Faranah (FA); Forécariah (FO); Gaoual (GA); Guéckédou (GU); Kindia (KD); Koundara (KN); Kouroussa (KO); Macenta (MC); Mamou (MM); Mandiana (MD) and N’zérékoré (NZ)). The number of samples by species are indicated above the pictograms with different colors (blue = cattle, green = goat, red = sheep). Map has been generated with QGIS software (version 3.18.1, https://www.qgis.org/en/site/)

**Fig. 2:**
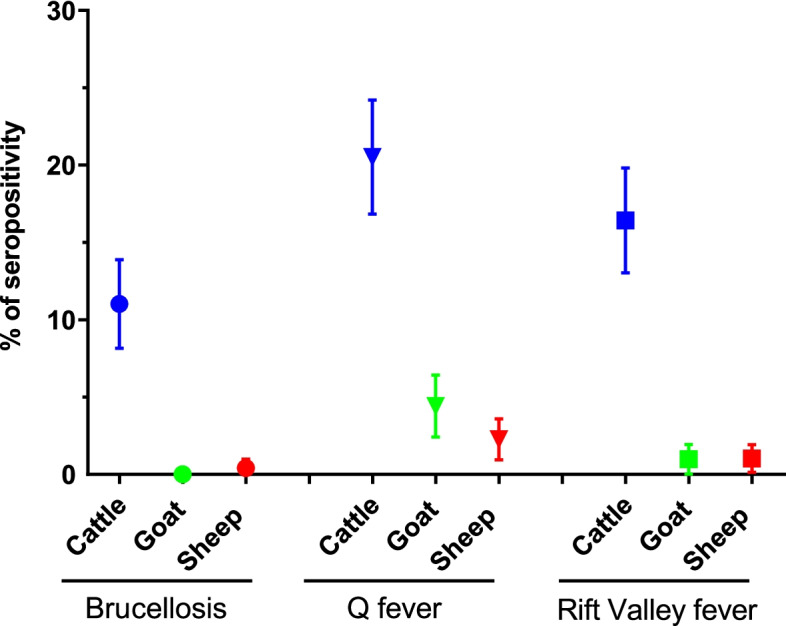
Seroprevalence of brucellosis, Q fever and Rift Valley fever in cattle, goats and sheep in Guinea. The apparent seroprevalences with the 95% confidence intervals are represented by the same color code as in Fig. 1 (blue = cattle, green = goat, red = sheep)

A total of 1357 samples (cattle = 463, goats = 408, sheep = 486) from 17 prefectures of Guinea (Fig. 1) were analysed for the presence of specific antibodies against *Brucella spp., C. burnetii* and Rift Valley fever virus (RVFV) using multi-species ELISA kits (IDVet). The overall seroprevalence was significantly different between the three diseases: *Brucella spp.* is 3.9% (95% confidence intervals (CI) 2.9—5.1); Q fever is 9.1% (95% CI 7.7—10.8), RVF is 6.3% (95% CI 5.1—7.7) (Table S2). Interestingly, seroprevalence in cattle for the three pathogens is 5 to 20-fold higher than in small ruminants (goat and sheep) (Table 1 and Fig. 2).

**Table 1:** Seroprevalence by species and pathogens

	Brucellosis		Q fever		Rift Valley fever	
Species	Tested (Positive)	Prevalence (95% CI)	Tested (Positive)	Prevalence (95% CI)	Tested (Positive)	Prevalence (95% IC)
Cattle	463 (51)	11.0 (8.3–14.2)	463 (95)	20.5 (16.9–24.5)	463 (76)	16.4 (13.2–20.1)
Goat	408 (0)	0 (0–0.9)	408 (18)	4.4 (2.6–6.9)	408 (4)	1.0 (0.3–2.5)
Sheep	486 (2)	0.4 (0.05–1.5)	486 (11)	2.3 (1.1–4.0)	486 (5)	1.0 (0.3–2.4)
**Total**	**1357 (53)**	**3.9 (2.9–5.1)**	**1357 (124)**	**9.1 (7.7–10.8)**	**1357 (85)**	**6.3 (5.0–7.7)**

### Seroprevalence of brucellosis

**Fig. 3:**
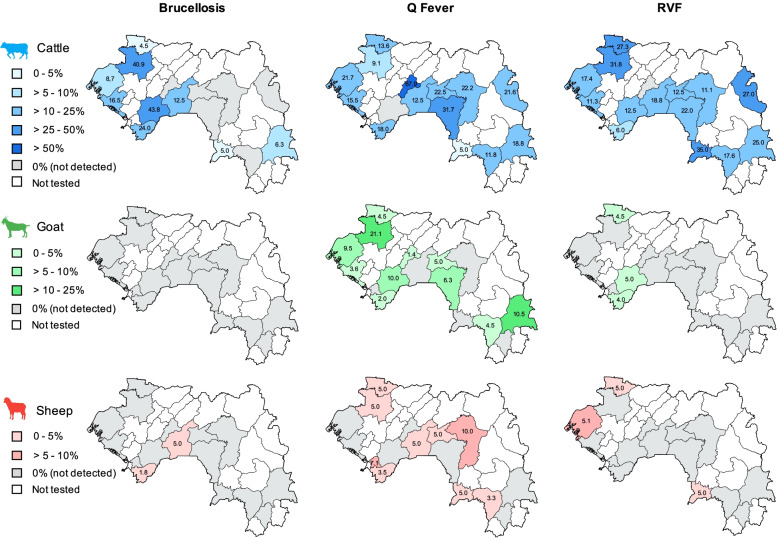
Maps of Guinea showing seroprevalence of brucellosis, Q fever and Rift Valley fever according to animal species and prefectures. The seroprevalence rate in each prefecture is indicated by a number in black according to a color code by animal species (blue for cattle, green for goat, red for sheep). A density color-scale highlights the seroprevalence level. The prefectures where no antibody was detected are in gray. Prefectures that were not investigated are in white. Maps have been generated with QGIS software (version 3.18.1, https://www.qgis.org/en/site/)

Antibodies to brucellosis were detected using ELISA in 51 cattle, 2 sheep and no goat resulting in a mean seroprevalence of 11.0% (95% CI 8.3—14.2), 0.4% (95% CI 0.05—1.5) and 0% (95% CI 0—0.9) respectively (Fig. 2 and Table 1). In cattle, seroprevalence was observed in 9 of the 15 (60%) prefectures visited, with highly variable values ranging from 4.5 to 43.8%. Overall, prefectures in West Guinea (Kindia-Gaoual and Forécariah-Boffa to a lesser extent) appeared to be more affected by brucellosis than prefectures in Central-East Guinea (Dabola, Dalaba, Faranah, Gueckedou, Koundara, Kouroussa, Macenta and Mandiana) (Fig. 3 and S1).

**Fig. 4:**
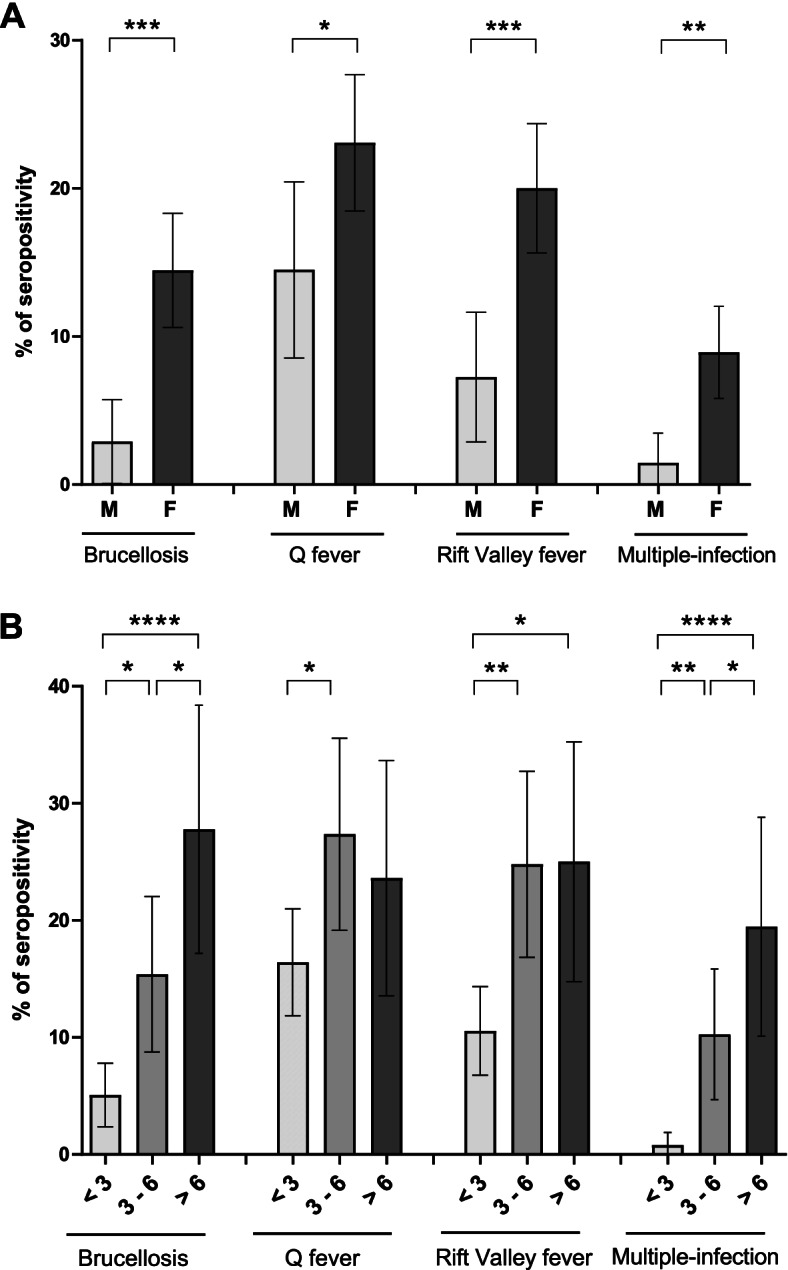
Influence of sex and age on seroprevalence of brucellosis, Q fever, Rift Valley fever and multiple-infection in cattle. **A** Seroprevalence of females and males with 95% confidence intervals are represented by histogram. *P*-values were determined according to the Mann–Whitney test (*p < 0.05; **p < 0.01; ***p < 0.001). **B** Seroprevalence in different age groups (in years) with 95% confidence intervals are represented by histogram. *P*-values were determined according to Kruskal–Wallis test followed by Dunn’s correction (*p < 0.05; **p < 0.01; ****p < 0.0001)

In cattle, seroprevalence of brucellosis was 5 times higher in females than in males (Fig. 4A). The risk of having contact with the pathogen was also increased with age: below 3 years of age it was 3 times and 5 times lower than those of 3–6 years of age and > 6 years of age, respectively (Fig. 4B). In contrast to the prevalence of brucellosis in cattle, only 2 out of 16 prefectures (12.5%) showed positive sheep and none out of 16 prefectures (0%) showed positive goats (Fig. 3). These low numbers made the statistical analysis by age and sex meaning less, however it is interesting to note that the two seropositive sheep were over 1-year old females (Fig. S2).

### Seroprevalence of Q fever

Q fever was the most frequently detected zoonosis among the samples tested, with a mean seroprevalence of 20.5% (95% IC 16.9—24.5), 4.4% (95% IC 2.6—6.9) and 2.3% (95% IC 1.1—4.0) in cattle, goats and sheep, respectively (Fig. 2 and Table 1). The prevalence of Q fever was detected in a large proportion of the prefectures visited: 14 out of 15 (93%) in cattle, 11 out of 16 (69%) in goats and 9 out of 16 (56%) in sheep with a strong seroprevalence distribution heterogeneity according to species and prefecture: 5.0 to 67.9% in cattle, 1.4 to 21.1% in goats and 3.3 to 10.0% in sheep (Fig. 3). Unlike brucellosis, no significant difference was found in the geographical distribution of Q fever, except that seroprevalence was significantly higher (2 to 5 times) in cattle in Dalaba compared to the 14 remaining prefectures (Fig. 3 and S1).

Similarly, seroprevalence in cattle was 1.5-fold higher in females than in males (Fig. 4A) and young cattle under 3 years of age were about 2 times less seropositive than adults (Fig. 4B). However, no significant difference was observed between adults aged 3–6 years and over 6 years, with the latter group even showing a slightly lower seroprevalence. On the other hand, sex and age had a less significant influence on Q fever seroprevalence in small ruminants (Fig. S2).

### Seroprevalence of Rift Valley fever

Antibodies to RVFV were detected by cELISA in 76 cattle, 4 goats and 5 sheep, resulting in a mean seroprevalence of 16.4% (95% CI 13.2—20.1), 1.0% (95% CI 0.3—2.5) and 1.0% (95% CI 0.3—2.4), respectively (Fig. 2). In cattle, this affected 14 out of the 15 prefectures visited (93%) and RVF seroprevalence is quite balanced between them, varying between 6.0 and 35.0%. In small ruminants, seroprevalence was found in only 3 out of the 16 prefectures (19%), with homogenous values ranging from 4.0 to 5.1% (Fig. 3 and S1).

In cattle, seroprevalence was 3 times higher in females than in males (Fig. 4A). Young cattle under 3 years of age showed a seroprevalence 2.5 times lower than cattle over 3-years old (Fig. 4B). The low number of positive sheep and goat prohibited statistical analysis by age and sex, however, it is interesting to note that the 5 seropositive sheep were females over 1-year old (Fig. S2).

To confirm that RVFV was circulating and not another phlebovirus, a sero-neutralisation test was performed on the serum of 5 ELISA-positive and 3 negative cattle. Neutralising antibodies were detected only in the 5 ELISA-positive cattle with titres > 1600.

### Seroprevalence for more than one NZD

Of the 1357 ruminants tested, 262 (19.3%) were seropositive for one of the three zoonoses, and 34 of them (2.5%) showed specific antibodies for more than one zoonotic agent: 9 cattle and 1 sheep were seropositive for both *Brucella spp.* and *C. burnetii,* 6 cattle for *Brucella spp.* and RVFV, 12 cattle, 1 goat and 1 sheep for *C. burnetii* and RVFV. Finally, 4 cattle (0.8%) were found seropositive for the three pathogens (Table 2). As expected, in cattle, the animals with serological traces of the three pathogens were mainly adult females (Fig. 4).

**Table 2:** Number of animals with serological traces of at least two pathogens

Associated diseases	Animal Number	Probability	Expected Animal Number	Observed Animal Number
**Bruc. + Q fev**				
Cattle	463	1.4–3.5%	6.5–16.2	9
Goat	408	0–0.06%	0–0.25	0
Sheep	486	0.001–0.06%	0.003–0.3	1
**Bruc. + RVF**				
Cattle	463	1.1–2.9%	5.1–13.25	6
Goat	408	0–0.02%	0–0.09	0
Sheep	486	0.0002–0.04%	0.001–0.2	0
**Q fev. + RVF**				
Cattle	463	2.2–4.9%	10.3–22.8	12
Goat	408	0.01–0.2%	0.03–0.7	1
Sheep	486	0.004–0.1%	0.02–0.5	1
**Bruc. + Q fev. + RVF**				
Cattle	463	0.2–0.7%	0.9–3.2	4
Goat	408	0–0.002%	0–0.01	0
Sheep	486	0–0.001%	0–0.01	0

The probability of multiple infection is calculated from the seroprevalence rate of each disease by species. The expected and observed number of successively or co-infected animals are indicated. Bruc. = brucellosis; Q fev. = Q fever; RVF = Rift Valley fever

## Discussion

This study provides, for the first time for Guinea, seroprevalence data on three major NZDs, i.e. brucellosis, Q fever and Rift Valley fever (RVF), conducted in a short-time period (2017 to mid-2019) on the same 1357 ruminants: 463 cattle, 408 goats and 486 sheep. Such an extensive comparison of the three NZDs in three different ruminant species has rarely been done in West Africa [3] and in Africa [32, 33]. Consistent with previous studies, we found higher overall seroprevalence of Q fever (9.1%) and RFV (6.3%) than brucellosis (3.9%). This difference in seroprevalence between the pathogens could be explained by their different modes of transmission.

In addition, seroprevalence was generally 5 to 20-fold higher in cattle than in goats and sheep. An obvious explanation is that cattle live longer than small ruminants and have more time/opportunity for infections, which then leads to long detectable antibodies. This is evident from the mean age of the available samples in animals: cattle (46.3 ± 3 months, range 3–216), goats (19.7 ± 1.5 months, range 2–72) and sheep (20.4 ± 1.6 months, range 1–156) (Table S1).

While Q fever was previously considered a rare and regionally restricted tropical disease in Africa (11), our results show that it circulates in many prefectures in Guinea. This is in agreement with a previous report of seroprevalence in cattle in Upper Guinea (6.6 ± 1.3%), Forest Guinea (7.8 ± 1.7%), Central Guinea (9.1 ± 1.0%) and Maritime Guinea (7.5 ± 1.3%) [28]. In the present study, new affected prefectures such as Boffa (15.5%), Boke (21.7%) and Gaoual (9.1%) were identified, indicating a geographical spread of Q fever in Guinea in recent years. This observation is also supported by a higher Q fever seroprevalence in cattle (20.5%) compared to the 2013 study (8.0 ± 0.6%). It would be important to re-evaluate in the coming years the seroprevalence of ruminants in the same areas to verify if this spreading tendency is continuous and to recommend adapted mitigation measures. Both the huge concentrations of *C. burnetii* present in amniotic fluids and placenta during parturition and the spore-like form highly resistant to heat and dry once in the environment can explain the rapid dissemination by wind for long distances.

In contrast, brucellosis is more prevalent in the West (Kindia-Gaoual-Forécariah-Boffa) than in Central-East Guinea. The reasons for this geographical discrepancy remain to be clarified but could be linked to the fact that the bacteria better survive in an environment with cool moist conditions as in West Coastal Guinea. Our results are consistent with the finding of two earlier studies: one from the 1980s, which reported seroprevalence in Kindia (7.4%), Coyah (13.3%) and Forécariah (20%), but not in Dalaba or Kankan [25] and a second from the early 2000s reporting seroprevalence in Boké (6.3%), Coyah (5.9%), Dubréka (12.7%), Forécariah (3.8%) but not in Labé [34]. We also found an overall prevalence in cattle (11.0%), which is within the range of previous studies (6.5% to 11.75%) [25–27, 31]. In contrast, one prefecture in East Guinea, namely Beyla, previously showed an atypically high brucellosis prevalence of 21.43% [27]. It is noteworthy that this prefecture is the only one in East Guinea where we found a low brucellosis seroprevalence of 6.3% (Fig. 3).

Regarding RVF in cattle, we found an overall seroprevalence of 16.4% (0 to 35.0%) that was significantly higher than in previous studies (0 to 6.8%) [27, 29, 30]. It is of note that prefectures known to practice intensive cattle breeding showed high seroprevalence (i.e. around 30% in Gaoual/Koundara). For risk mitigation, it would be important to perform seasonal sampling in these areas to link the dynamics of serological occurrence to the circulation of RVFV in relevant insect vectors. Vector distribution is highly dependent from changes in climate and land use, weather conditions and water availability, human mobility and animal trade increasing the opportunity for vectors to establish in new areas. The RVF seroprevalence was significantly lower (around 1%) in small ruminants, but reached 5% in some prefectures, i.e. Boké, Forécariah, Guéckédou, Kindia and Koundara as previously observed [31].

An interesting finding of the present study is that several animals were found seropositive for more than one of the three NZDs suggesting that they have been successively or co-infected by the corresponding pathogens. Globally, the number of animals seropositive for more than one disease is statistically coherent based on the seroprevalence of each infection suggesting minimal influence between infections (Table 1). However, there may be competition between *C. burnetii* and *Brucella sp*. infections in cattle. In the prefectures of Dabola, Dalaba, Faranah, Kouroussa and Mandiana, where Q fever prevalence is higher (> 20%), brucellosis prevalence is null. In Kindia and Gaoual prefectures, where brucellosis prevalence is higher (> 40%), Q fever prevalence is null or low. The reason for this potential interference resulting in bi-directional antagonistic effects should be more precisely investigated. Specific immune cross-protection is improbable due to the genetic distance between both bacteria. Competition for the same ecological niche in term of infection or transmission has to be explored. It must be also noted that both bacteria (specially for *Brucella sp.)* are able to survive for several months in the environment [4]. Finally, Dalaba prefecture is the only one where the circulation of RFV has not been detected. Although the cooler climate could explain the rarity of RVFV vectors (i.e. *Aedes sp.* and *Culex sp.),* it is noteworthy that Dalaba has the highest rate of Q fever.

Up to now, most studies on brucellosis seroprevalence in Africa have been focused in cattle only [25–27, 35]. Our study confirms a significant seroprevalence in Guinean cattle (51 positives; 11.0%) but no seropositive goats and only 2 sheep. This difference between cattle and small ruminants was already observed in Western Africa and Ethiopia and could result from a more active circulation of *B. abortus* rather than *B. melitensis* in Guinea [3, 6, 33, 36, 37]. Indeed in Western Africa almost 90% of isolates are *B. abortus* [35] while *B. melitensis* has been associated with brucellosis epidemics in small ruminants mainly in Northern, Eastern and Central African countries [6, 35, 38]. In this context, it would be interesting in the future to isolate and identify which *Brucella spp.* are circulating among the ruminants in Guinea.

RVF seroprevalence studies in African ruminants have delivered contradictory results [23]. Most of them show similar seroprevalence levels between cattle and small ruminants [3, 39–43] or higher seroprevalence in cattle [33, 44–48]. Only few reports show higher seroprevalence in goats and sheep [49, 50]. The sensitivity of different animal species to RVFV infection is another subject of controversy and several reports indicated similarity between cattle and small ruminants. One study in Madagascar has suggested that cattle are more attractive for RVFV mosquito vectors [51] whereas an epizootic in Kenya in 2006–2007 showed that RVFV mosquito vectors such as *Aedes ochraceus*, *Aedes mcintoshi*, and *Mansonia uniformis* have feeding preference for goats, followed by cattle, donkeys, sheep, and humans [52]. Entomological studies are required in Guinea to define the host preference of RVF mosquito vectors. Indeed, sheep and goats, with a faster population turnover than cattle, offer a bigger pool of susceptible individuals [23, 47]. They are generally relevant sentinel species for RVF outbreaks with a seroprevalence up to 100% during some epizootics [23, 24]. In this context, the low prevalence in small ruminants that we observed in Guinea could indicate that the samples have been collected during interepidemic period for RVF.

Consensus exists that Q fever seroprevalence is higher in goat and sheep is than in cattle [53]. This is confirmed in Africa by other studies in Chad, Egypt, Ethiopia and Kenya [32, 33, 37, 54, 55] while other reported similar Q fever seroprevalence between ruminant species [3, 36, 56]. Only few studies showed a higher cattle seroprevalence as we found in Guinea [57, 58]. This could be explained by a higher exposition period for cattle compared to small ruminant [33, 58, 59].

Our study indicates that in cattle, females have higher risk to be infected than males by Q fever (1,5 -fold), RVF (threefold) and Brucellosis (fivefold). Similar results were previously reported across Africa [33, 36] and particularly in Chad with 2, 3 and fourfold, respectively [46]. One straightforward explanation is the lifespan as previously argued to explain the seroprevalence differences between cattle and small ruminants. In our cattle sampling, the mean age of male (29.9 ± 3.3 months ranging from 2 to 124) was significantly lower than that of female (53.1 ± 3.8 months, ranging from 6 to 216). In addition, for brucellosis and Q fever, huge concentrations of *C. burnetii* and *Brucella spp.* are found in amniotic fluid and placenta. Finally, female can be infected by *Brucella spp.* several times during life from parturition time with boost at each new birth. This explains that cattle seroprevalence is lower under 3 years old and steadily increase with age [33]. In addition, it has been shown for brucellosis that males are able to eliminate specific antibodies and to become seronegative.

## Conclusions

Although targeting only asymptomatic animals, the present study highlights the circulation of three abortive animal diseases in Guinea with occurrence of simultaneous or consecutive co-infection. This invites further studies to investigate the mechanisms of interference between infections by the longitudinal follow up of selected farms from different ecological area in Guinea. Our results also invites the veterinary services to reinforce syndromic surveillance of ruminant abortions. Collection of vaginal swabs, placenta and/or abortive fetus could allow to better identify the infectious agents of these three diseases and to differentiate them from other pathogens provoking abortion such as *Tritrichomonas foetus*, *Camphylobacter spp*. or Bovine Viral Diarrhea virus. Multiplex serological (Luminex) or genetic (multiplex PCR, resequencing chips) tools encompassing the main suspected aetiological agents should be developed in this perspective. Moreover, given the high bovine seroprevalence level of brucellosis, Q fever and RFV in some Guinean prefectures, it will be important to set up a One Health approach assessing the incidence of these zoonotic diseases in human and investigating the routes of transmission, in particular in people having close contacts with cattle such as breeders, veterinarians and/or slaughterers.

## Methods

### Sampling process

A total of 1357 blood samples (cattle = 463, goats = 408, sheep = 486) from 17 prefectures of Guinea were included in this study (Fig. 1). To cover a large geographical area, samples from three different collections were used: i) 698 samples (cattle = 205, goats = 201, sheep = 292) were collected by the Institut Pasteur de Guinée (IPGui) between October 2017 and June 2019; ii) 228 samples (cattle = 128, goats = 50, sheep = 50) by IRD (Institut de Recherche pour le Développement) and PNLTHA (Programme National de Lutte contre la Trypanosomose Humaine Africaine) as part of research programmes on the animal reservoir of trypanosomiasis between February 2017 and April 2019 and iii) 431 samples (cattle = 130, goats = 157, sheep = 144) was collected by the Laboratoire Central Vétérinaire de Diagnostic (LCVD) from the Guinean Ministry of Animal Husbandry as part of an FAO study on brucellosis conducted between October and November 2019. IPGui's and IRD sampling strategy were reviewed by the Guinean Comité National d'Ethique pour la Recherche en Santé (CNERS) under number 040/CNERS/17 and 102/CNERS/19 respectively. Sampling campaigns were organized with the consent of the local veterinary authorities and breeders were informed of the purpose of the study and provided signed informed consent. All sampled animals were randomly selected from different herds in which animals were considered healthy with no correlation with any symptoms. Age and sex information was recorded for each animal based on information provided by breeders and visual verification of sex and approximate age by collaborating veterinarians (Table S1). Among the 1357 samples the age and sex information were available for 1255 and 1356 animals respectively.

Blood samples were collected from animals using 5 mL dry vacutainer tubes and kept at cool temperature (4 to 8 °C) and centrifuged for 10 min at 3 000 RPM as rapidly as possible in the field to avoid hemolysis. Sera were stored frozen (-20 °C in the vehicle, -80 °C in the laboratory) until the serological tests were carried out.

### Serological tests

Before serological analysis all sera were inactivated at 56 °C for 30 min. Sera were run in three different multi-species Enzyme-Linked Immunosorbent Assays (ELISA) from IDVet for detection of specific antibodies against *Brucella sp., C. burnetii* and RVFV: *ID Screen Brucellosis Serum Indirect Multi-Species*, *ID Screen Q-Fever Indirect Multi-Species* and *ID Screen Rift Valley Fever Competition Multi-Species*, respectively. ELISA tests were performed and validated according to the manufacturer’s protocols and the test results were interpreted as positive, doubtful or negative. All sera giving positive or doubtful results in the first ELISA were retested a second time using the same ELISA kit. Only samples confirmed positive by the second test were considered positive, all the others were considered negative.

### RVFV neutralization assay

Neutralizing antibodies (Ab) against RVFV were measured as previously described in BSL3 conditions [60, 61]. Sera were serially diluted 1:100 to 1:1600 in DMEM and incubated with 100 pfu of the MP-12 strain of RVFV at 37^◦^C with 5% CO_2_ for 1 h. Next, the virus/serum mix was inoculated onto VeroE6 cell monolayers in 12-well-plates (5 × 10^5^ cells per well). One hour after adsorption at 37 °C, 2 mL of diluted carboxymethylcellulose sodium salt with DMEM supplemented with 5% FBS (v/v) were added to each well and plates were incubated at 37 °C in 5% CO_2_ for 5 days. The plaque forming units (pfu) were then revealed by 1% (w/v) crystal violet and the number of pfu/mL was calculated for each serum. The neutralizing Ab titers were established as the last dilution which inhibited 50% of the foci number per well compared to virus-only control titration.

### Data analysis

Data were analyzed with the GraphPad Prism 6.0 software. The unpaired non-parametric Mann–Whitney test was used to compare female and male seroprevalence for each disease and species. The non-parametric Kruskal–Wallis test followed by Dunn’s correction was used to compare the seroprevalence among age groups and prefectures 2 by 2 for each diseases and species.

## Supplementary Information

**Additional file 1.** 

## Data Availability

The datasets used and/or analyzed during the current study are available from the corresponding author on reasonable request.

## Abbreviations

CNERS: Comité National d'Ethique pour la Recherche en Santé / National Ethical Comittee for Health Research
ELISA: Enzyme-linked immunosorbent assay
FAO: Food and Agriculture Organization of United Nations
IPGui: Institut Pasteur de Guinée / Institute Pasteur of Guinea
IRD: Institut de Recherche pour le Développement / French Research Institut for Development
LCVD: Laboratoire Central Vétérinaire de Diagnostic / Central Veterinary laboratory of diagnosis
PNLTHA: Programme National de Lutte contre la Trypanosomose Humaine Africaine / National African Human Trypanosomiasis Control Program
NZD: Neglected Zoonotic Disease
PFU: Plaque forming units
RVF: Rift Valley fever
RVFV: Rift Valley fever virus
