# Supplementary material for: Seroprevalence of brucellosis, Q fever and Rift Valley fever in domestic ruminants in Guinea in 2017–2019
Source: BMC Vet Res. 2022 Feb 4;18:64. doi: 10.1186/s12917-022-03159-x (PMC8815129; doi:10.1186/s12917-022-03159-x)
Supplement: Supplementary file 1 — Additional file 1. [file 12917_2022_3159_MOESM1_ESM.pdf]

Prevalence of brucellosis, Q fever and Rift Valley fever in domestic ruminants in Guinea in 2017-2019.

Supplementary files

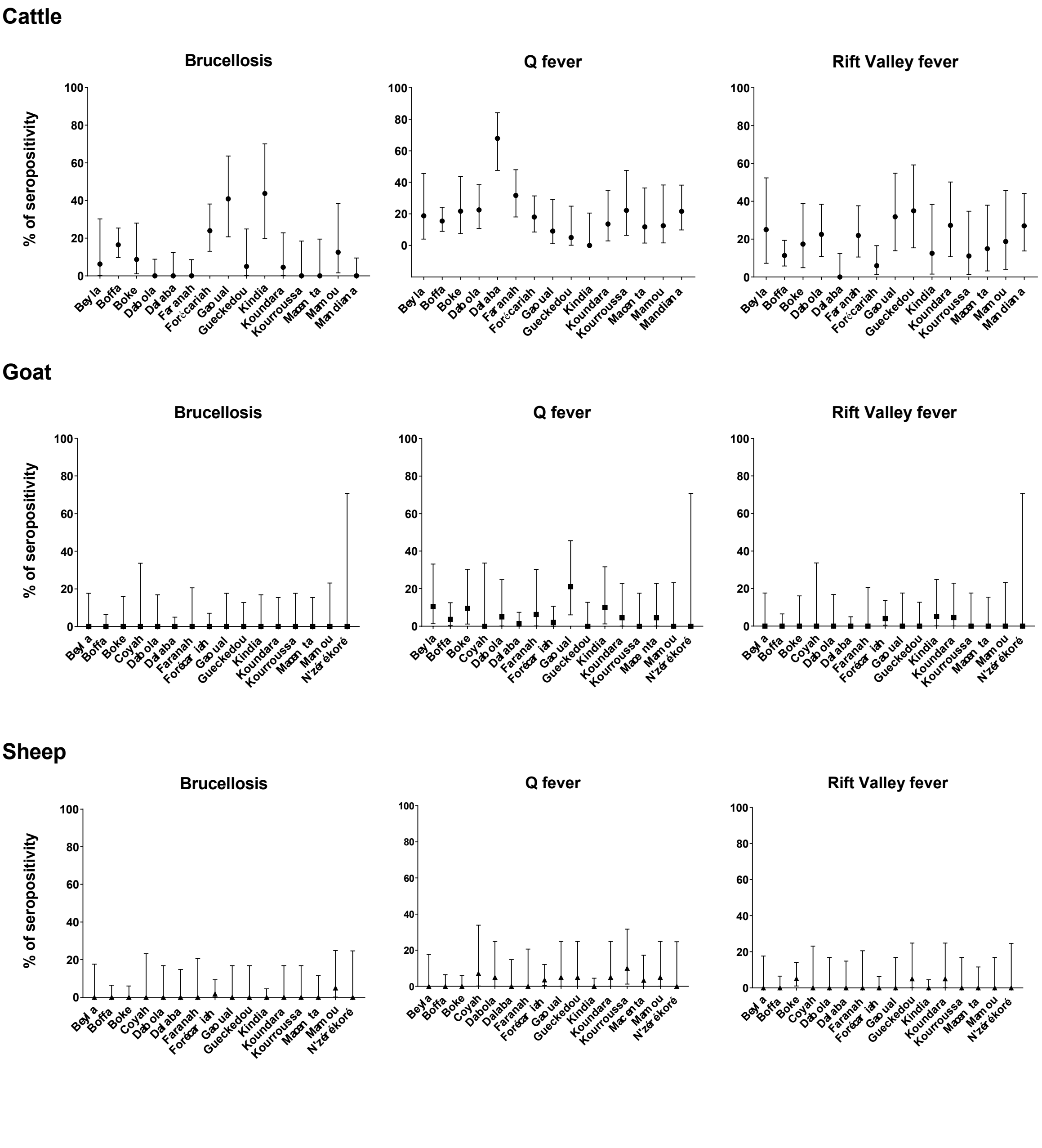

Fig S1. Seroprevalence of brucellosis, Q fever and Rift Valley fever according to animal species and prefectures. The apparent seroprevalences with the 95% confidence intervals are represented on the graphs.

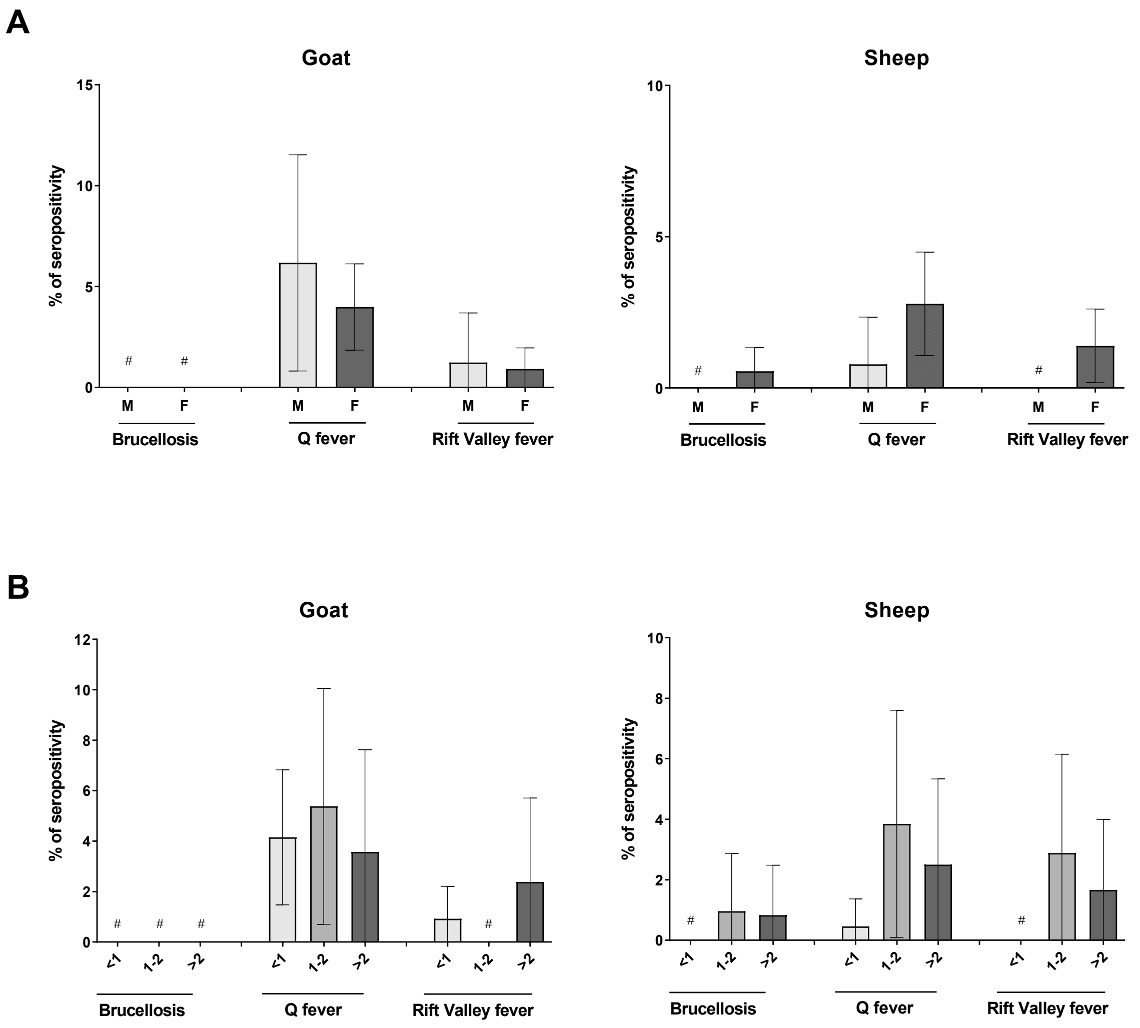

Fig S2. Influence of sex and age on seroprevalence of brucellosis, Q fever, Rift Valley fever in small ruminants. Histograms represent: A) Seroprevalence of females and males with 95% confidence intervals; B) Seroprevalence in different age groups (in years) with 95% confidence intervals. [#]: no animal was available in this group.

Table S1. Age (in months) and sex characteristics of sampled animals

Species	Animal numbers	Age mean (range)	Animal number by age groups			
			0 - 36# 0 - 12$	37 - 72# 13 - 24$	> 72# > 24$	NI
Cattle						
Male	138	29,92 (3 – 124)	108	21	2	7
Female	325	53,13 (6 – 216)	148	96	70	11
Total	463	46,73 (3 – 216)	256	117	72	18
Goat						
Male	81	13,43 (3 – 36)	53	15	8	5
Female	326	21,24 (2 – 72)	139	78	75	34
Total	408*	19,68 (2 – 72)	192	93	84*	39
Sheep						
Male	127	13,14 (2 – 60)	85	18	13	11
Female	359	23,03 (1 – 156)	132	86	107	34
Total	486	20,33 (1 – 156)	217	104	120	45

NI: not informed; #: for cattle; $: for goat and sheep; *: sex information missing for 1 goat
